# Efficacy and safety of surufatinib plus toripalimab, a chemotherapy-free regimen, in patients with advanced gastric/gastroesophageal junction adenocarcinoma, esophageal squamous cell carcinoma, or biliary tract cancer

**DOI:** 10.1007/s00262-024-03677-7

**Published:** 2024-05-07

**Authors:** Panpan Zhang, Zhendong Chen, Si Shi, Zhiping Li, Feng Ye, Lijie Song, Yanqiao Zhang, Fei Yin, Xing Zhang, Jianming Xu, Ying Cheng, Weiguo Su, Michael Shi, Songhua Fan, Panfeng Tan, Chen Zhong, Ming Lu, Lin Shen

**Affiliations:** 1 Key Laboratory of Carcinogenesis and Translational Research (Ministry of Education/Beijing), Department of Early Drug Development Centre, Peking University Cancer Hospital and Institute, Beijing, 100142 China; 2 Department of Oncology, The Second Affiliated Hospital of Anhui Medical University, No.678 Furong Road, Economic and Technological Development Zone, Hefei, Anhui China; 3 Department of Pancreatic Hepatobiliary Surgery, Fudan University Shanghai Cancer Center, No.270 Dong’an Road, Xuhui District, Shanghai, China; 4 Department of Abdominal Oncology, West China Hospital of Sichuan University, No.37 Guoxue Lane, Wuhou District, Chengdu, Sichuan China; 5 Department of Medical Oncology, The First Affiliated Hospital of Xiamen University, No.55 Zhenhai Road, Siming District, Xiamen, Fujian China; 6 First Department of Oncology, The First Affiliated Hospital of Zhengzhou University, No.1 East Jianshe Road, Erqi District, Zhengzhou, Henan China; 7 Second Department of Gastroenterology, Harbin Medical University Cancer Hospital, No.150 Haping Road, Nangang District, Harbin, Heilongjiang China; 8 Department of Gastroenterology, The Fourth Hospital of Hebei Medical University, No.12 Jiankan Road, Shijiazhuang, Hebei China; 9 Biotherapy Center, Sun Yat-sen University Cancer Center, No.651 East Dongfeng Road, Yuexiu District, Guangzhou, Guangdong China; 10 Department of Gastrointestinal Oncology, The Fifth Medical Center of Chinese PLA General Hospital, No.8 East Avenue, Fengtai District, Beijing, China; 11 Department of Thoracic Oncology, Jilin Cancer Hospital, No.1066 Jinghu Avenue, Gaoxin District, Changchun, Jilin China; 12 HUTCHMED Limited, Building 4, 720 Cailun Road, Pilot Free Trade Zone, Shanghai, China; 13 State Key Laboratory of Holistic Integrative Management of Gastrointestinal Cancers, Beijing Key Laboratory of Carcinogenesis and Translational Research, Department of Gastrointestinal Oncology, Peking University Cancer Hospital and Institute, Beijing, 100142 China

**Keywords:** Gastric cancer, Esophageal squamous cell carcinoma, Biliary tract carcinoma, Surufatinib, Toripalimab

## Abstract

**Background:**

The programmed death 1 inhibitor toripalimab plus the angio-immuno kinase inhibitor surufatinib showed a tolerable safety profile and preliminary efficacy in patients with advanced solid tumors in a phase I study.

**Methods:**

This open-label, multi-cohort study in China enrolled patients with advanced solid tumors who had failed or were intolerable to standard treatment into tumor-specific cohorts. Patients received surufatinib (250 mg orally, once daily) plus toripalimab (240 mg intravenously, once every three weeks). Results for three cohorts (gastric/gastroesophageal junction [GC/GEJ] adenocarcinoma, esophageal squamous cell carcinoma [ESCC], and biliary tract carcinoma [BTC]) are reported here. The primary endpoint was investigator-assessed objective response rate (ORR) per Response Evaluation criteria in Solid Tumors version 1.1.

**Results:**

Between December 17, 2019, and January 29, 2021, 60 patients were enrolled (GC/GEJ, *n* = 20; ESCC, *n* = 20; BTC, *n* = 20). At data cutoff (February 28, 2023), ORRs were 31.6%, 30.0%, and 11.1%, respectively. Median progression-free survival was 4.1, 2.7, and 2.9 months, respectively. Median overall survival was 13.7, 10.4, and 7.0 months, respectively. Overall, grade ≥  3 treatment-related adverse events occurred in 28 (46.7%) patients.

**Conclusions:**

Surufatinib plus toripalimab showed promising antitumor activity and a tolerable safety profile in immunotherapy-naïve patients with GC/GEJ adenocarcinoma, ESCC, or BTC. These findings warrant further study in larger randomized trials comparing surufatinib plus toripalimab with standard therapies in these tumors.

*ClinicalTrials.gov* NCT04169672.

**Supplementary Information:**

The online version contains supplementary material available at 10.1007/s00262-024-03677-7.

## Introduction

For advanced/metastatic unresectable solid tumors related to the gastrointestinal (GI) system, cytotoxic chemotherapy, used either with or without immune checkpoint inhibitors (ICIs), is the recommended first-line treatment. However, persistent toxicities from initial treatment with cytotoxic chemotherapy can limit the ability of many patients with progressive disease to receive a second-line therapy, particularly in gastric/gastroesophageal junction (GC/GEJ) adenocarcinomas [1]. In addition, second-line therapy is associated with modest efficacy. For patients with advanced esophageal squamous carcinoma (ESCC), the efficacy of programmed cell death protein 1 (PD-1) monotherapy remained limited: overall survival (OS) was ≤  9 months and median progression-free survival (PFS) was not prolonged compared with chemotherapy [2, 3]. For patients with relapsed advanced biliary tract carcinoma (BTC), both chemotherapy and targeted therapies showed limited benefit [4]. After multiple lines of treatments, many patients have poor physical status and resistance to chemotherapy is common. Thus, developing a chemotherapy-free regimen is an unmet need.

While ICIs have shown clinical benefits in multiple solid tumors [5, 6], many patients eventually progressed after treatment with ICI monotherapy. Antiangiogenic therapies targeting vascular endothelial growth factor (VEGF) or VEGF receptor-2 (VEGFR-2) have been shown to promote trafficking of T cells into tumors and modulate suppressive immune cells, which could help to overcome resistance to ICIs [7, 8]. Combination therapy with a VEGF/VEGFR inhibitor plus PD-1 inhibitor has shown efficacy in various tumors such as renal clear cell carcinoma, advanced endometrial cancer, and unresectable hepatocellular carcinoma, respectively [9, 10].

In a phase I trial, surufatinib, an oral small-molecule inhibitor which targets VEGFRs 1, 2, and 3, fibroblast growth factor receptor 1, and colony stimulating factor 1 receptor [11], combined with toripalimab, a recombinant humanized monoclonal immunoglobulin G4, anti–PD-1 antibody [12, 13], was well tolerated by patients with advanced solid tumors and showed encouraging signs of antitumor activity [12]. Among 30 patients with advanced solid tumors enrolled in the study, objective response rate was 24.1%. Most treatment-related adverse events (TRAEs) in this study were mild or moderate, with no TRAE leading to treatment discontinuation. The most common grade ≥  3 TRAEs were hypertension (20.0%), transaminases increased (13.3%), and blood bilirubin increased (13.3%). The RP2D was determined to be 250 mg orally, once daily, for surufatinib and 240 mg intravenously, once every three weeks, for toripalimab, used as a combination therapy.

We further performed an exploratory, basket, multi-cohort study to further evaluate the activity of a chemotherapy-free regimen, surufatinib plus toripalimab, in patients with advanced solid tumors who had failed or were intolerable to standard treatment. The results for the GC/GEJ adenocarcinoma, ESCC, and BTC cohorts are shown here.

## Methods

### *Study design and participants*

Eligible participants were adults (aged 18–75 years) with histologically or cytologically confirmed, unresectable or metastatic advanced solid tumors who had failed (≤ 1 lines of systemic chemotherapy for GC/GEJ, ESCC, BTC) or were intolerant to standard treatment or for whom there is currently no effective therapy. Additional eligibility criteria included an Eastern Cooperative Oncology Group performance status (ECOG PS) of 0 or 1, at least one measurable lesion (per Response Evaluation Criteria in Solid Tumors version 1.1 [RECIST v1.1]), availability of a biopsy sample (fresh or archival) for detection of PD-L1 expression level, and adequate organ function. Patients who had received prior treatment with immune checkpoint inhibitors were excluded. A full account of the study eligibility criteria is shown in Supplementary Table 1.

### *Procedures*

All patients received combination treatment with surufatinib (250 mg orally, once daily) plus toripalimab (240 mg intravenously, once every three weeks). Patients continued with treatment until disease progression (PD), death, unacceptable toxicity, patient withdrawal, or loss to follow-up (whichever occurred first), but for a maximum duration of 24 months with toripalimab.

Tumors were assessed by investigators per RECIST v1.1 and immune-related RECIST (irRECIST) every six weeks from the first dose to 48 weeks, and then every 12 weeks thereafter. Evaluation of tumors was performed using standard imaging modalities according to tumor type; bone scan was used in patients with suspected bone metastases. Objective complete responses (CRs) or partial responses (PRs) were confirmed four weeks after the first observation. Patients’ survival was assessed every 12 weeks from study treatment discontinuation until patient death, loss to follow-up, withdrawal of informed consent, or end of study (whichever occurred first).

Safety was assessed by recording the incidence, severity, and investigator-assessed causality of treatment-emergent adverse events and serious adverse events (SAEs), which were collected within 90 days from the first dose until the last dose of study drug or before initiation of a subsequent antitumor therapy, whichever occurred first. Sponsor-assessed immune-related adverse events (irAEs) related to toripalimab were also recorded. All adverse events were coded based on MedDRA version 25.1, and the severity was graded according to the National Cancer Institute Common Terminology Criteria for Adverse Events version 5.0.

Immunohistochemistry for detection of programmed death-ligand 1 (PD-L1) expression in tumor samples was performed using the Ventana SP263 rabbit monoclonal primary antibody on the BenchMark ULTRA System (Roche, AZ, USA). PD-L1 expression was measured as combined positive score (CPS), defined as the number of PD-L1–stained cells (tumor cells, lymphocytes, macrophages) divided by the total number of viable tumor cells, multiplied by 100 (maximum score, CPS 100).

### *Outcomes*

The primary endpoint was ORR per RECIST v1.1. Secondary endpoints were ORR by irRECIST, duration of response (DoR), PFS, disease control rate (DCR) by both RECIST v1.1 and irRECIST, and overall survival (OS). Definitions of all endpoints are shown in Supplementary Table 2. Initial complete response (CR) or partial response (PR) was confirmed again after ≥ 4 weeks. Safety endpoints included incidence and severity of TRAEs and change from baseline in targeted vital signs, clinical laboratory test results, and ECOG PS.

### *Statistical analysis*

The sample size for the study was based on feasibility. A total of 260 patients with evaluable advanced solid tumors were planned for enrollment (of which, three cohorts are reported in this manuscript), with 10–20 in each of the solid tumor cohorts.

The primary analyses (ORR, DCR) were based on the efficacy-evaluable analysis set (EEAS), which referred to all patients who had received at least one dose of the study drug and had at least one valid post-baseline tumor assessment. Supportive analyses were conducted for the full analysis set (FAS) population—all patients who received at least one dose of the study drug. DoR was analyzed based on patients with confirmed CR or PR. Primary analyses of baseline characteristics, safety, PFS, and OS were conducted for the FAS population.

ORR, DCR, and corresponding exact 95% confidence intervals (CIs) were calculated using the Clopper–Pearson method. Median and 95% CI values were estimated for DoR, PFS, and OS using Kaplan–Meier statistics. Subgroup analyses according to tumor PD-L1 expression were also conducted. All statistical analyses were performed using SAS Enterprise Guide 8.3 (64-bit). This trial is registered with ClinicalTrials.gov (NCT04169672).

## Results

### Patient characteristics

Between December 17, 2019, and January 29, 2021, 60 of the 84 screened patients were enrolled into three cohorts and treated across 11 sites in China (GC/GEJ *n* = 20, ESCC *n* = 20, and BTC *n* = 20; Supplementary Fig. 1). At the time of data cutoff (February 28, 2023), all (100%) patients across the three cohorts had permanently discontinued from the study treatment, most commonly due to radiographic PD (*n* = 38, 63.3%), followed by withdrawal by patient (*n* = 12, 20.0%) and adverse event (*n* = 3, 5.0%) (Supplementary Fig. 1).

The demographic characteristics were generally consistent across the three tumor cohorts (Table 1). All patients were Asian and median age ranged from 58 to 65 years across cohorts. The majority of patients had stage IV disease (*n* = 56, 93.3%) and had received just one prior anticancer therapy (*n* = 53, 88.3%). The most frequently reported anticancer medication was platinum drugs and fluorouracil drugs (*n* = 19, 95.0% for each) in the GC/GEJ cohort, platinum drugs (*n* = 19, 95.0%) in the ESCC cohort, and gemcitabine and fluorouracil drugs (*n* = 13, 65.0% for each) in the BTC cohort, respectively (Supplementary Table 3). PD-L1 expression levels varied across the cohorts (Table 1).

**Table 1:** Baseline demographic and disease characteristics

	GC/GEJ	ESCC	BTC
	(*n* = 20)	(*n* = 20)	(*n* = 20)
*Age, years*			
Mean (SD)	60.2 (8.2)	60.8 (7.2)	61.7 (9.5)
Median	58	60	65
IQR	54–67	56–66	59–66
*Sex*			
Male	16 (80.0)	14 (70.0)	10 (50.0)
Female	4 (20.0)	6 (30.0)	10 (50.0)
*ECOG PS*			
0	4 (20.0)	2 (10.0)	9 (45.0)
1	16 (80.0)	18 (90.0)	11 (55.0)
*Tumor stage at screening*			
III	2 (10.0)	1 (5.0)	1 (5.0)
IV	18 (90.0)	19 (95.0)	19 (95.0)
*No. of involved organs*			
1	6 (30.0)	3 (15.0)	2 (10.0)
2	7 (35.0)	11 (55.0)	8 (40.0)
≥ 3	7 (35.0)	6 (30.0)	10 (50.0)
*PD-L1 CPS*			
< 5	11 (55.0)	7 (35.0)	12 (60.0)
≥ 5 to < 10	3 (15.0)	5 (25.0)	6 (30.0)
≥ 10	6 (30.0)	8 (40.0)	1 (5.0)
Missing	0	0	1 (5.0)
*Prior anticancer therapies*			
0	1 (5.0)	2 (10.0)	1 (5.0)
1	19 (95.0)	15 (75.0)	19 (95.0)
2	0	3 (15.0)	0

Data are *n* (%) unless otherwise stated

*BTC* biliary tract cancer, *CPS* combined positive score, *ECOG PS* Eastern Cooperative Oncology Group performance score, *ESCC* esophageal squamous cell carcinoma, *GC* gastric, *GEJ* gastroesophageal junction, *IQR* interquartile range, *SD* standard deviation

### Efficacy

**Fig. 1:**
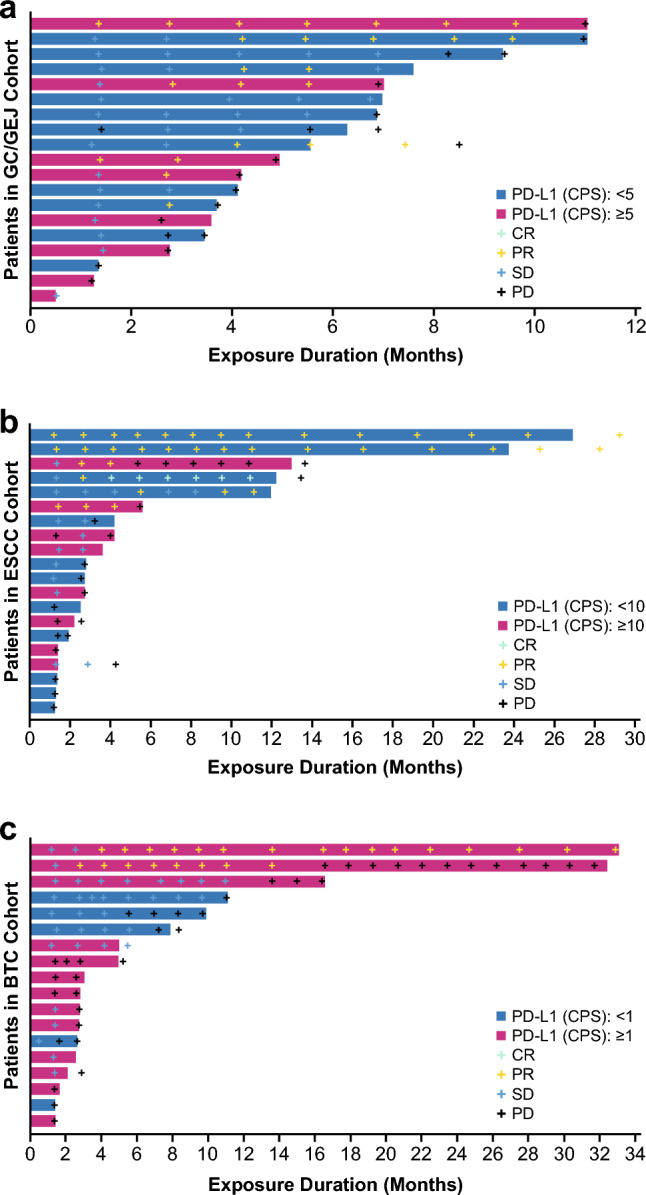
Tumor response and treatment duration, by RECIST v1.1

**Fig. 2:**
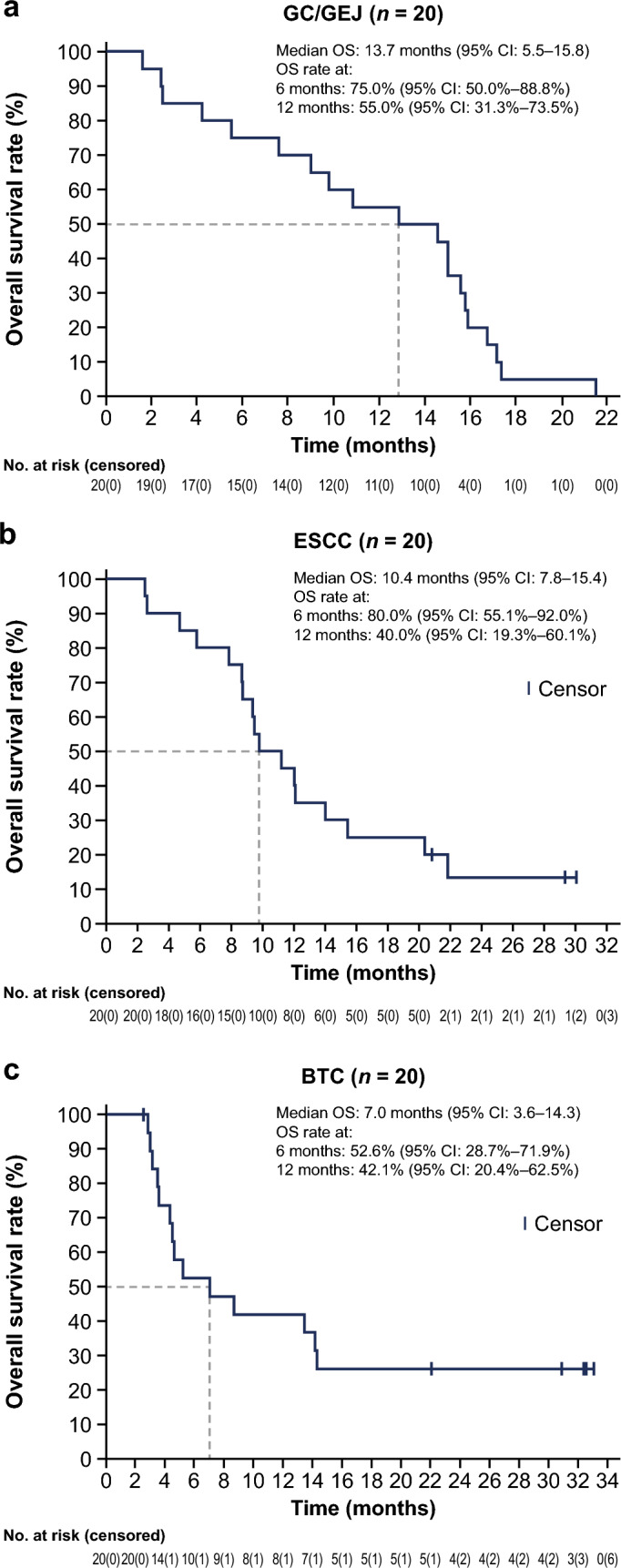
Kaplan–Meier estimates of overall survival by tumor cohort

As the study design involved multiple tumor types and each malignancy differs in characteristics, efficacy data were presented for each individual cohort (Table 2, Figs. 1 and 2, Supplementary Table 4 and 5) rather than for the combined analysis. Overall, the primary efficacy endpoint of ORR by RECIST v1.1 (Table 2) yielded similar results as those assessed by irRECIST (Supplementary Table 5), as did other key secondary endpoints including DoR, DCR, and PFS (Supplementary Table 5). The subgroup analysis of efficacy by PD-L1 expression for selected cohorts is shown in Supplementary Table 6.

**Table 2:** Efficacy data by tumor type, by RECIST v1.1

	GC/GEJ	ESCC	BTC
EEAS, *n*	(*n* = 19)	(*n* = 20)	(*n* = 18)
Best overall response, *n* (%)			
Complete response	0	1 (5.0)	0
Partial response	6 (31.6)	5 (25.0)	2 (11.1)
Stable disease	9 (47.4)	6 (30.0)	9 (50.0)
Disease progression	3 (15.8)	8 (40.0)	7 (38.9)
Not evaluable	1 (5.3)	0	0
Objective response rate, *n* (%)	6 (31.6)	6 (30.0)	2 (11.1)
95% CI^a^	12.6 to 56.6	11.9 to 54.3	1.4 to 34.7
Disease control rate, *n* (%)^b^	15 (78.9)	12 (60.0)	11 (61.1)
95% CI^a^	54.4 to 94.0	36.1 to 80.9	35.8 to 82.7
Duration of response^c^			
Median, months	4.3	8.7	-^d^
95% CI^c^	3.4 to NE	2.8 to NE	13.8 to NE

*BTC* biliary tract cancer, *EEAS* efficacy-evaluable analysis set, *ESCC* esophageal squamous cell carcinoma, *GC* gastric, *GEJ* gastroesophageal junction, *NE* not evaluable, *RECIST v1.1* Response Evaluation Criteria in Solid Tumors version 1.1

^a^95% CIs are based on Clopper–Pearson exact confidence interval

^b^Disease control rate = complete response + partial response + stable disease

^c^Median and 95% CI are based on the Kaplan–Meier method

^d^At data cutoff, only one patient in BTC cohort with an objective response had experienced tumor progression. The duration of response of this patient was 13.8 months

In the GC/GEJ cohort, a best tumor response of PR was achieved in six out of 19 evaluable patients in the EEAS (one patient was not evaluable for efficacy); nine had stable disease (SD). ORR was 31.6% (95% CI: 12.6–56.6) and DCR was 78.9% (95% CI: 54.4–94.0). Median DoR was 4.3 months (95% CI: 3.4–not evaluable [NE]), median PFS was 4.1 months (95% CI: 2.6–7.6), and median OS was 13.7 months (95% CI: 5.5–15.8).

Among patients with ESCC (*n* = 20 in the EEAS), one patient achieved CR; a further five and six patients had PR and SD, respectively, for an ORR of 30.0% (95% CI: 11.9–54.3) and a DCR of 60.0% (95% CI: 36.1–80.9). Median DoR was 8.7 months (95% CI: 2.8–NE). Median PFS and OS were 2.7 months (95% CI 1.3–5.5) and 10.4 months (95% CI: 7.8–15.4), respectively.

Two of 18 evaluable patients in the BTC cohort achieved a best tumor response of PR, and nine (50.0%) had SD. The corresponding ORR and DCR were 11.1% (95% CI: 1.4–34.7) and 61.1% (95% CI: 35.8–82.7), respectively. Of the two patients with objective response, only one patient had PD as of data cutoff date, with a DoR of 13.8 months. Median PFS was 2.9 months (95% CI: 1.4–7.2) and median OS was 7.0 months (95% CI: 3.6–14.3).

### Safety

TRAEs (either study drug) of any grade occurred in 58 (96.7%) patients (Table 3). Mean duration of exposure ranged from 5.3 to 7.4 months across cohorts. The most frequently occurring TRAEs were proteinuria (*n* = 30, 50.0%), occult blood positive (*n* = 23, 38.3%), diarrhea (*n* = 21, 35.0%), blood bilirubin increased (*n* = 18, 30.0%), and urinary occult blood positive (*n* = 18, 30.0%; Supplementary Table 4). Grade ≥ 3 TRAEs were reported in 28 (46.7%) patients, with the most common events being hypertension (*n* = 4, 6.7%), neutrophil count decreased (*n* = 3, 5.0%), and white blood cell count decreased (*n* = 3, 5.0%; Supplementary Table 7). Seventeen (28.3%) patients reported treatment-related SAEs. TRAEs led to surufatinib dose modification in 29 (48.3%) patients and toripalimab interruption in 12 (20.0%) patients. TRAEs leading to discontinuation of either study drug occurred in six (10.0%) patients (Table 3), and the events were all a single occurrence. Treatment-related adverse events led to death in two (3.3%) patients, including immune-mediated lung disease in one patient with ESCC, and death in one patient with GC, with an unknown cause due to confounders such as disease progression or underlying disease.

**Table 3:** TRAEs for the intent-to-treat population

Summary of TRAEs, *n* (%)	GC/GEJ	ESCC	BTC	Total
	(*n* = 20)	(*n* = 20)	(*n* = 20)	(*N* = 60)
Any TRAEs	20 (100.0)	19 (95.0)	19 (95.0)	58 (96.7)
Grade ≥ 3	8 (40.0)	11 (55.0)	9 (45.0)	28 (46.7)
Surufatinib-related adverse events	20 (100.0)	18 (90.0)	19 (95.0)	57 (95.0)
Grade ≥ 3	7 (35.0)	11 (55.0)	9 (45.0)	27 (45.0)
Leading to surufatinib dose interruption or dose reduction	9 (45.0)	11 (55.0)	9 (45.0)	29 (48.3)
Toripalimab-related adverse events	18 (90.0)	18 (90.0)	18 (90.0)	54 (90.0)
Grade ≥ 3	4 (20.0)	4 (20.0)	6 (30.0)	14 (23.3)
Leading to toripalimab interruption	4 (20.0)	5 (25.0)	3 (15.0)	12 (20.0)
Leading to discontinuation of either study drug	2 (10.0)	2 (10.0)	2 (10.0)	6 (10.0)
Leading to discontinuation of surufatinib	2 (10.0)	2 (10.0)	1 (5.0)	5 (8.3)
Leading to discontinuation of toripalimab	1 (5.0)	1 (5.0)	2 (10.0)	4 (6.7)
Leading to death	1 (5.0)	1 (5.0)	0	2 (3.3)
Treatment-related SAE	4 (20.0)	7 (35.0)	6 (30.0)	17 (28.3)
irAE related to toripalimab	8 (40.0)	13 (65.0)	10 (50.0)	31 (51.7)
Grade ≥ 3	1 (5.0)	2 (10.0)	2 (10.0)	5 (8.3)

*irAE* immune-related adverse event, *TRAE* treatment-related adverse event

Toripalimab-related irAEs occurred in 31 (51.7%) patients and were grade ≥  3 in five (8.3%) (Table 3). irAEs of grade ≥  3 reported in ≥  2 patients included immune-related diabetes (*n* = 2, 3.3%) and immune-related hepatitis (*n* = 2, 3.3%).

## Discussion

This exploratory study showed that surufatinib plus toripalimab yielded encouraging antitumor activity and an acceptable safety profile in patients with advanced GC/GEJ adenocarcinoma, ESCC, or BTC who had failed or were intolerable to standard treatment.

Across the three cohorts, ORRs ranged from 11.1 to 31.6%, with the highest seen in the GC/GEJ and ESCC cohorts (31.6% and 30.0%, respectively). There were also encouraging signs of disease control; DCRs were high (≥ 60.0%) across all cohorts, with the highest (78.9%) in the GC/GEJ cohort; median OS ranged from 7.0 to 13.7 months. Although the median PFS was relatively short in all three cohorts (median PFS 2.7–4.1 months), there appear to be signs of benefits in terms of objective responses and OS. In preclinical tumor models, surufatinib combined with anti–PD-1 or anti–PD-L1 could reverse immunosuppressive tumor microenvironment by inhibiting tumor angiogenesis, increasing activated cytotoxic T-cell infiltration, elevating the ratio of CD8 + T/Treg and reducing M2 tumor associated macrophages [14]. We hypothesize that the combination of surufatinib plus toripalimab may affect the tumor immune microenvironment and remodel its sensitivity to subsequent chemotherapy, which may explain the resulting short PFS but relative long OS observed in our study, wherein at least 76.7% of patients had received prior chemotherapy, and at least 24 in 33 patients who had received subsequent antitumor therapy after discontinuation of study drug had received subsequent chemotherapy. Nonetheless, further studies are required to validate this.

Currently, approved second-line therapies for advanced/metastatic unresectable GC include ramucirumab monotherapy, ramucirumab plus paclitaxel, docetaxel, or irinotecan, with ORR ranging between 0 and 28% and median OS ranging between 4.0 and 9.6 months [15–19]. However, the outcome for the second-line setting remains poor. In KEYNOTE-061, pembrolizumab did not significantly improve OS compared with paclitaxel as second-line therapy for metastatic GC with PD-L1 CPS ≥ 1 (median OS was 9.1 months with pembrolizumab and 8.3 months with paclitaxel) [20]. In our study, response was particularly noteworthy in patients with GC/GEJ adenocarcinoma, with an ORR of 31.6% and DCR of 78.9%; median PFS was 4.1 months and OS was 12.9 months, which appeared favorable compared with the standard second-line treatments. These encouraging response rates and survival data are particularly intriguing when considered in the context of previous trials with toripalimab given as a monotherapy to patients with GC/GEJ. In a phase Ib/II study, toripalimab monotherapy yielded an ORR of 12.1% and DCR of 39.7% in patients with advanced gastric cancer who were refractory to chemotherapy; median PFS was 1.9 months and OS was 4.8 months [21]. Further exploration in a larger sample size is warranted.

The ORR in the ESCC cohort was relatively high at 30.0% (DoR 8.7 months) and included the only patient in the study who achieved CR; median OS was 10.4 months. These findings are comparable with the current National Comprehensive Cancer Network–recommended second-line treatments for advanced ESCC, such as nivolumab, pembrolizumab, and camrelizumab, which showed ORRs ranging from 16.7 to 20.2% and median OS ranging from 6.8 to 10.9 months [2, 3, 22, 23].

For the BTC cohort, the ORR of 11.1% and DCR of 61.1% was particularly promising compared with FOLFOX, the current recommended second-line therapy for metastatic unresectable BTC (ORR 5%, DCR 33%) [24]. While, there is currently a lack of high-quality conclusive evidence to support immunotherapy in the second-line treatment of advanced BTC, the favorable antitumor activity demonstrated by surufatinib plus toripalimab indicates that this combination therapy could offer a potential chemotherapy-free regimen if these findings are further validated in larger studies.

The combination of surufatinib plus toripalimab demonstrated an acceptable safety profile. Rates of any or grade ≥ 3 TRAEs for surufatinib and toripalimab were consistent with those observed in prior trials of surufatinib or toripalimab monotherapy, with no unexpected safety signals [11, 13, 25]. In general, TRAEs were manageable with supportive care, dose modification, and treatment interruption and/or discontinuation. TRAEs leading to discontinuation of either study drug were generally singular occurrences. Treatment-related SAEs were reported in 26.3% of patients, of which decreased platelet count were reported in two patients in the ESCC cohort; the others were all single occurrence in each cohort. Most of these events, such as decreased platelet count, anemia, and myelosuppression, were manageable and did not substantially impact quality of life. In addition, the safety profile was similar to a phase I trial using the same combination in patients with advanced solid tumors [12].

The relevance of this study is limited by recent phase III data establishing chemotherapy plus a checkpoint inhibitor as first-line therapy for GC/ESCC/BTC. It should be noted that there is a change in the contemporary first-line treatment for GC and ESCC during the duration of our study, whereby immunotherapy was placed as an important first-line therapy. For patients with HER2-negative GC, nivolumab plus fluoropyrimidine and oxaliplatin is one of the recommended first-line therapies for patients with PD-L1 CPS ≥ 5 [26]. Although most patients in our study received chemotherapy as the first-line treatment, patients with PD-L1 CPS < 5 (*n* = 11) still derived additional clinical benefits from surufatinib plus toripalimab in the second-line setting: ORR 27.3%, DCR 81.8%, DoR 4.4 months, median PFS 6.9 months, and median OS 15.0 months (Supplementary Table 7). Similar findings were also observed in the ESCC cohort. Pembrolizumab in combination with platinum- and fluoropyrimidine-based chemotherapy is recommended for patients with ESCC with PD-L1 CPS ≥ 10 as first-line treatment (median OS 13.5 months) [27]. In our study, surufatinib plus toripalimab conferred potential survival benefit in patients with PD-L1 CPS < 10 (*n* = 7), whereby the median OS was 12.0 months (Supplementary Table 7). This indicates that surufatinib plus toripalimab may be a potential novel chemotherapy-free regimen for patients with ESCC, particularly those with CPS < 10, for whom there is currently no approved treatment. Further study with a larger population and longer period is needed to validate these findings.

While a basket study design provides a great opportunity to assess the efficacy and safety of a novel treatment regimen across diverse types of tumors, it may increase the risk that cohorts could be too small to detect meaningful outcomes. The limitations of this trial are typical of early phase studies, including the small sample size in each tumor cohort and the open-label, single-arm study design. The sample size was determined based on feasibility without statistical assumptions, as the primary purpose of this study was to explore the potential efficacy of surufatinib plus toripalimab in any specific type of tumor. The responses and safety profile observed in each of the present tumor cohorts would need to be verified in larger populations and in randomized controlled studies. In addition, the findings may not be generalizable to other populations as this study only enrolled Chinese patients.

In conclusion, the chemotherapy-free regimen of surufatinib plus toripalimab, delivered predominantly as a second-line therapy, showed promising antitumor activity, with a manageable safety profile in immunotherapy-naïve patients with advanced GC/GEJ adenocarcinoma, ESCC, or BTC. Larger studies are warranted to validate these findings.

### Supplementary Information

Below is the link to the electronic supplementary material.

Supplementary file1 (DOCX 168 KB)

## Data Availability

The authors confirm that the data supporting the findings of this study are available within the article or its supplementary materials.

## Abbreviations

BTC: Biliary tract carcinoma
CR: Complete response
CI: Confidence interval
DCR: Disease control rate
DoR: Duration of response
ECOG PS: Eastern Cooperative Oncology Group performance status
EEAS: Efficacy-evaluable analysis set
ESCC: Esophageal squamous cell carcinoma
FAS: Full analysis set
GC/GEJ: Gastric/gastroesophageal junction
GI: Gastrointestinal
ICIs: Immune checkpoint inhibitors
irAEs: Immune-related adverse events
irRECIST: Immune-related RECIST
NE: Not evaluable
NETs: Neuroendocrine tumors
ORR: objective response rate
OS: Overall survival
PD: Disease progression
PR: Partial response
PD-1: Programmed cell death protein 1
PD-L1: Programmed death-ligand 1
PFS: Progression-free survival
RECIST v1.1: Response Evaluation Criteria in Solid Tumors version 1.1
SAEs: Serious adverse events
TRAEs: Treatment-related adverse events
VEGF: Vascular endothelial growth factor
VEGFR-2: Vascular endothelial growth factor receptor-2
