# Supplementary material for: Efficacy and safety of surufatinib plus toripalimab, a chemotherapy-free regimen, in patients with advanced gastric/gastroesophageal junction adenocarcinoma, esophageal squamous cell carcinoma, or biliary tract cancer
Source: Cancer Immunol Immunother. 2024 May 7;73(7):119. doi: 10.1007/s00262-024-03677-7 (PMC11076424; doi:10.1007/s00262-024-03677-7)

Supplemental Material

# Supplemental Methods

***Table 1:* Study eligibility criteria**

| **Inclusion criteria** |
| --- |
| 1. Have fully understood and voluntarily signed the informed consent form for this study (the informed consent form must be signed before any trial-specific procedures are performed); |
| 1. Aged 18-75 years (inclusive); |
| 1. Patients with histologically or cytologically confirmed unresectable or metastatic advanced solid tumors including biliary tract cancer, gastric adenocarcinoma and gastroesophageal junction adenocarcinoma, esophageal squamous cell carcinoma and other tumors; |
| 1. Patients who have failed standard treatment (disease progression after treatment or intolerable toxic and side effects of treatment), have no standard treatment or cannot receive standard treatment. |
|  |
| **Surufatinib in combination with toripalimab treatment group**: the main tumor types included biliary tract cancer, gastric adenocarcinoma and gastroesophageal junction adenocarcinoma and esophageal squamous cell carcinoma:   - - Gastric adenocarcinoma and gastroesophageal junction adenocarcinoma: patients with progressive disease or intolerable toxic and side effects after receiving first-line chemotherapy (containing fluorouracil and platinum);   - Biliary tract cancer (including biliary/extrahepatic cholangiocarcinoma and gallbladder cancer): patients with progressive disease or intolerable toxic and side effects after first-line systemic chemotherapy (based on gemcitabine or fluorouracil);   - Esophageal squamous cell carcinoma: patients with progression or intolerance to first-line systemic chemotherapy (may include platinum, paclitaxel or fluorouracil-based); |
| 1. ECOG performance status of 0 or 1; |
| 1. Definite measurable lesions meeting the requirements of RECIST (1.1); if the lesion previously treated with local therapy (radiotherapy, ablation, vascular intervention, etc.) is the only lesion, there must be a clear imaging basis for the disease progression of the lesion; |
| 1. Agreement on providing tissue examination specimens for the detection of PD-L1 expression level (only applicable to combination therapy group), microsatellite instability (MSI) or mismatch repair gene (MMR) detection. MSI and MMR are only applicable to patients with endometrial cancer, and further confirm the pathological grade when necessary; |
| 1. Adequate bone marrow, liver and kidney organ function, laboratory tests within 7 days prior to the first dose meeting the following requirements:    1. Absolute neutrophil count (ANC) ≥ 1.5 × 10^9^/L, platelets ≥ 100 × 10^9^/L and hemoglobin ≥ 90 g/L (no blood transfusion, no blood products, no granulocyte colony-stimulating factor or other hematopoietic stimulating factor correction 14 days before laboratory tests); 2. Serum total bilirubin ≤ 1.5 × upper limit of normal reference range (× ULN); 3. Alanine aminotransferase (ALT) and aspartate aminotransferase (AST) ≤ 1.5 × ULN in the absence of liver metastases; ALT and AST ≤ 3 × ULN in the presence of liver metastases; 4. Serum creatinine ≤ 1.5 × ULN and creatinine clearance ≥ 50 mL/min (calculated according to Cockcroft-Gault formula); 5. Urinalysis shows urine protein <2 +; if urine protein ≥ 2 +, 24-hour urine protein quantification should be <1 g; 6. International normalized ratio (INR) ≤ 1.5 and activated partial thromboplastin time (APTT) ≤ 1.5 × ULN |
| 1. Life expectancy ≥ 12 weeks; |
| 1. Women of childbearing potential must have a negative serum pregnancy test within 7 days prior to the first dose. Male or female patients of childbearing potential voluntarily use effective contraceptive methods during the study and within 90 days after study drug administration, such as double-barrier contraceptive methods, condoms, oral or injectable contraceptives, intrauterine devices, etc. All female patients are considered to be of childbearing potential unless they are postmenopausal, have undergone artificial menopause, or have undergone surgical sterilization (eg, hysterectomy, surgical adnexectomy). |
| **Exclusion criteria** |
| 1. Previous anticancer treatment-related toxicities not recovered to CTCAE grade ≤ 1, except alopecia and oxaliplatin-induced peripheral neurotoxicity CTCAE grade ≤ 2; |
| 1. Other malignancies within the past 5 years (except for basal cell carcinoma or squamous cell carcinoma of the skin that has been effectively controlled, cervical carcinoma in situ); |
| 1. Patients with symptomatic central nervous system (CNS) metastasis or carcinomatous meningitis (LM) during the screening period; patients who have been treated for CNS metastasis and clinically stable 2 weeks before the first dose, have discontinued corticosteroids or reduced the dose of systemic corticosteroids to ≤ 10 mg/day prednisone (or equivalent) for at least 2 weeks could be enrolled; |
| 1. Received systemic anti-tumor therapy that are approved within 4 weeks prior to the first medication, including: chemotherapy (oral fluoropyrimidines washout period of 2 weeks), biological therapy, targeted therapy (small molecule targeted drugs washout period of 2 weeks or 5 half-lives, whichever is shorter), hormone therapy, traditional Chinese medicine therapy (traditional Chinese medicine treatment with clear anti-tumor indications in the instructions, after a 1-week washout period before the first medication); |
| 1. Received radical radiotherapy (including more than 25% bone marrow radiotherapy) within 4 weeks prior to the first dose; brachytherapy (such as implantation of radioactive seeds) within 60 days before the first dose; received palliative radiotherapy for bone metastases 1 week before the first dose; |
| 1. Patients who previously have received treatment with any anti-PD-1 antibody, anti-PD-L1 antibody, anti-PD-L2 antibody or anti-cytotoxic T-lymphocyte-associated antigen-4 (CTLA-4) antibody, or any other antibody acting on T cell co-stimulation or checkpoint pathway, such as OX40, CD137, etc. (this exclusion criterion is only applicable to combination therapy group) or Surufatinib therapy, except for patients who previously received immunotherapy as specified in the protocol; |
| 1. Patients who previously have received anti-VEGF/VEGFR-targeted drug therapy and showed disease progression during treatment or within 4 months after treatment; |
| 1. Patients with thyroid dysfunction who are symptomatic or require treatment at screening (only hypothyroidism controlled by thyroid hormone replacement therapy can be included); |
| 1. Use of immunosuppressive agents within 4 weeks prior to first dose, excluding topical glucocorticoids for nasal spray, inhalation or other routes of administration or physiological doses of systemic glucocorticoids (ie, no more than 10 mg/day prednisone or equivalent doses of other glucocorticoids); temporary use of glucocorticoids for the treatment of asthma, chronic obstructive pulmonary disease and other dyspnea is allowed. This exclusion criterion only applies to the combination arm; |
| 1. Presence of any active autoimmune disease requiring systemic treatment or history of autoimmune disease within the past 2 years, including but not limited to interstitial pneumonia, uveitis, inflammatory bowel disease, hepatitis, hypophysitis, vasculitis, systemic lupus erythematosus, etc. (patients with vitiligo, psoriasis, alopecia or Grave’s disease not requiring systemic treatment within the past 2 years, and patients with type I diabetes only requiring insulin replacement therapy can be included); known history of primary immunodeficiency; patients with positive autoimmune antibodies need to confirm the presence of autoimmune disease at the investigator’s discretion. This exclusion criterion applies only to the combination treatment group; |
| 1. Received systemic immunostimulant therapy within 4 weeks prior to the first dose. This exclusion criterion applies only to the combination treatment group; |
| 1. Administration of any live or attenuated live vaccines within 4 weeks prior to the first dose or planned administration during the study; |
| 1. Patients who have undergone major surgery (refer to grade 3 and 4 surgeries specified in Management Measures for Clinical Application of Medical Technology on May 1, 2009 for the definition of major surgery) or have not healed wounds and fractures with clinical significance; or patients who are expected to undergo major surgery (except for surgeries for the purpose of diagnosis). Note: Implantation of central venous access catheters (eg, ports or similar devices) is permitted; |
| 1. Uncontrolled malignant pleural effusion, ascites or pericardial effusion (defined as not effectively controlled by diuretics or paracentesis, as judged by the investigator); |
| 1. Patients with hypertension uncontrolled by medication, defined as: systolic blood pressure ≥ 140 mmHg and/or diastolic blood pressure ≥ 90 mmHg; |
| 1. The patient has any disease or condition that affects drug absorption, or the patient cannot take the drug orally; |
| 1. Patients who have received strong CYP3A inducers or inhibitors within 1 week or 5 half-lives (whichever is longer) prior to the first dose; |
| 1. Patients with active gastric and duodenal ulcer, ulcerative colitis and other gastrointestinal diseases or unresected tumor with active bleeding, or other conditions that may cause gastrointestinal bleeding and perforation as judged by the investigator; |
| 1. Patients with evidence or history of significant bleeding tendency within 2 months before the first dose (bleeding >30 mL, with hematemesis, melena, hematochezia within 2 months), hemoptysis (fresh blood >5 mL within 4 weeks); |
| 1. Arterial thrombosis or deep venous thrombosis within 6 months or stroke and/or transient ischemic attack within 12 months prior to the first dose; |
| 1. Patients with clinically significant cardiovascular disease, including but not limited to acute myocardial infarction, severe/unstable angina or coronary artery bypass grafting within 6 months before the first dose; congestive heart failure of New York Heart Association (NYHA) class ≥ 2; left ventricular ejection fraction (LVEF) <50%; |
| 1. Clinically significant electrolyte abnormalities as judged by the investigator; |
| 1. Patient has an active infection or unexplained fever (temperature >38.5ºC) during screening, prior to first dose; |
| 1. Patients with active pulmonary tuberculosis (TB) who are receiving anti-tuberculosis treatment or have received anti-tuberculosis treatment within 1 year prior to the first dose; |
| 1. Patients with previous and current history of pulmonary fibrosis, interstitial pneumonia, pneumoconiosis, radiation pneumonitis, drug-related pneumonia, severely impaired pulmonary function and other diseases that may interfere with the detection and treatment of suspected drug-related pulmonary toxicity; radiation pneumonitis in the radiation therapy area is allowed; |
| 1. Positive human immunodeficiency virus (HIV) antibody screening; |
| 1. Known history of clinically significant liver disease, including active viral hepatitis [HBV DNA >1 × 10^3^ copies/mL or >200 IU/mL when positive for hepatitis B virus surface antigen (HBsAg) and/or hepatitis B virus core antibody (HbcAb); known positive hepatitis C virus (HCV) antibody and HCV RNA >1 × 10^3^ copies/mL], or other hepatitis, clinically significant moderate to severe cirrhosis; |
| 1. Received other clinical drug treatment that has not been approved or marketed in China within 4 weeks before the first medication; |
| 1. Women who are pregnant (positive pregnancy test before medication) or breastfeeding; |
| 1. Known hypersensitivity to any component of the toripalimab, surufatinib preparations, or previous history of significant allergy to any other monoclonal antibody; |
| 1. Patients who, in the opinion of the investigator, have other reasons that make them unsuitable for this clinical study |

***Table 2:* Definition of efficacy parameters**

CR=complete response; DCR=disease control rate; DoR=duration of response; ORR=objective response rate; OS=overall survival; PD=progressive disease; PFS=progression-free survival; PR=partial response; SD=stable disease.

| **Efficacy parameters** | **Definition** |
| --- | --- |
| Tumor response | |
| Objective response rate (ORR), % | Proportion of patients with confirmed CR or PR |
| Disease control rate (DCR), % | Proportion of patients with confirmed CR, PR, or SD |
| Duration of response (DoR), months | Time from the occurrence of a CR or PR (whichever occurred first) to PD or death |
| Survival | |
| Progression-free survival (PFS), months | Time from the first dose of the study drug to the first observation of tumor progression or death (prior to PD) |
| Overall survival (OS), months | Time from the first dose of the study treatment to death |

***Table 3: Summary of prior anti-cancer medication (*≥ 3 patients in any cohort)**

|  | **GC/GEJ**  **(N=20)**  **n (%)** | **ESCC**  **(N=20)**  **n (%)** | **BTC**  **(N=20)**  **n (%)** |
| --- | --- | --- | --- |
| **Prior anti-cancer medication** | **20 (100.0)** | **19 (95.0)** | **19 (95.0)** |
| Platinum drugs^1^ | 19 (95.0) | 19 (95.0) | 7 (35.0) |
| Fluorouracil drugs^2^ | 19 (95.0) | 3 (15.0) | 13 (65.0) |
| Paclitaxel^3^ | 5 (25.0) | 16 (80.0) | 2 (10.0) |
| Targeted therapeutic drugs^4^ | 1 (5.0) | 3 (15.0) | 1 (5.0) |
| Gemcitabine | 0 | 0 | 13 (65.0) |

Note:

1. Platinum drugs included oxaliplatin, cis-platinum, carboplatin, dedaplatin, and lobaplatin.
2. Fluorouracil drugs included fluorouracil, capecitabine, tegafur, and tegafur, gimeracil and oteracil potassium.
3. Paclitaxel included paclitaxel, albumin-bound paclitaxel, liposome taxol, and docetaxel.
4. Targeted therapeutic drugs included afatinib, apatinib mesylate, cetuximab, and trastuzumab.

***Table 4: Summary of survival data by tumor type, with tumor assessments performed by the investigator using RECIST v1.1***

|  | GC/GEJ | ESCC | BTC |
| --- | --- | --- | --- |
| FAS, n | 20 | 20 | 20 |
| PFS^‡^ |  |  |  |
| Median, months | 4.1 | 2.7 | 2.9 |
| 95% CI^‡^ | (2.6-7.6) | (1.3-5.5) | (1.4-7.2) |
| PFS rate at 3 months, % | 63.2 | 45.0 | 36.8 |
| 95% CI^‡^ | (37.9-80.4) | (23.1-64.7) | (16.5-57.5) |
| PFS rate at 6 months, % | 42.1 | 20.0 | 30.7 |
| 95% CI^‡^ | (20.4-62.5) | (6.2-39.3) | (12.1-51.7) |
| PFS rate at 9 months, % | 10.5 | 20.0 | 24.6 |
| 95% CI^‡^ | (1.8-28.4) | (6.2-39.3) | (8.1-45.6) |
| Median follow-up duration,^‡^ months | NE | 28.8 | 32.9 |
| 95% CI | (NE-NE) | (28.3-NE) | (4.2-NE) |
| OS^‡^ |  |  |  |
| Median, months | 13.7 | 10.4 | 7.0 |
| 95% CI^‡^ | (5.5-15.8) | (7.8-15.4) | (3.6-14.3) |
| OS rate at 6 months, %  95% CI^‡^  OS rate at 9 months, %  95% CI^‡^  OS rate at 12 months, % | 75.0  (50.0-88.8)  65.0  (40.3-81.5)  55.0 | 80.0  (55.1-92.0)  65.0  (40.3-81.5)  40.0 | 52.6  (28.7-71.9)  42.1  (20.4-62.5)  42.1 |
| 95% CI^‡^ | (31.3-73.5) | (19.3-60.1) | (20.4-62.5) |
| Median follow-up duration,^‡^ months | NE | 29.3 | 32.4 |
| 95% CI | (NE-NE) | (20.8-NE) | (22.1-NE) |

Note: Data for PFS and OS are based on the FAS.

BTC, biliary tract cancer; EEAS, efficacy-evaluable analysis set; ESCC, esophageal squamous cell carcinoma; GC, gastric; GEJ, gastroesophageal junction; FAS, full analysis set; NE, not evaluabe; OS, overall survival; PFS, progression-free survival; RECIST v1.1, Response Evaluation Criteria in Solid Tumors version 1.1.

^‡^Median and 95% CI are based on the Kaplan–Meier method.

***Table 5:* Summary of efficacy data by tumor type, with tumor assessments performed by the investigator using irRECIST [2]**

Data for best overall response, objective response rate, and disease control rate, and duration of response are based on the efficacy evaluable set; data for progression-free survival and overall survival are based on the FAS population. *95% CIs are based on Clopper-Pearson exact confidence interval. †Disease control rate=complete response+partial response+stable disease. ^§^Median and 95% CI are based on the Kaplan-Meier method. BTC=biliary tract carcinoma; EEAS=efficacy-evaluable analysis set; ESCC=esophageal squamous cell carcinoma; GC=gastric adenocarcinoma; GEJ=gastroesophageal junction carcinoma; irRECIST=Immune-Related Response Evaluation Criteria in Solid Tumors; FAS=full analysis set; NE=not evaluable.

|  | GC/GEJ | ESCC | BTC |
| --- | --- | --- | --- |
| EEAS, n | 19 | 20 | 18 |
| Best overall response, n (%) |  |  |  |
| Complete response | 0 | 1 (5.0) | 0 |
| Partial response | 6 (31.6) | 5 (25.0) | 2 (11.1) |
| Stable disease | 10 (52.6) | 9 (45.0) | 10 (55.6) |
| Disease progression | 2 (10.5) | 5 (25.0) | 6 (33.3) |
| Not evaluable | 1 (5.3) | 0 | 0 |
| Objective response rate, n (%) | 6 (31.6) | 6 (30.0) | 2 (11.1) |
| 95% CI* | (12.6-56.6) | (11.9-54.3) | (1.4-34.7) |
| Disease control rate, n (%)† | 16 (84.2) | 15 (75.0) | 12 (66.7) |
| 95% CI* | (60.4-96.6) | (50.9-91.3) | (41.0-86.7) |
| Duration of response^§^ |  |  |  |
| Median, months | 4.3 | 8.7 | NE |
| 95% CI* | (3.4-NE) | (2.8-NE) | (13.8-NE) |
| FAS, n | 20 | 20 | 20 |
| Progression-free survival,^§^ |  |  |  |
| Median, months | 4.9 | 3.2 | 2.9 |
| 95% CI | (2.7-7.6) | (1.4-5.5) | (1.4-7.2) |
| PFS rate at 3 months, %  95% CI*  PFS rate at 6 months, %  95% CI*  PFS rate at 9 months, % | 68.4  (42.8-84.4)  42.1  (20.4-62.5)  10.5 | 53.6  (29.6-72.6)  21.4  (6.7-41.6)  21.4 | 42.1  (20.4-62.5)  30.7  (12.1-51.7)  24.6 |
| 95% CI* | (1.8-28.4) | (6.7-41.6) | (8.1-45.6) |
| Median follow-up duration,^§^ months | NE | 28.3 | 32.9 |
| 95% CI | (NE-NE) | (28.3-NE) | (4.2-NE) |

***Table 6:* Summary of efficacy data (RECIST v1.1) by combined positive score for the gastric/gastroesophageal junction adenocarcinoma and esophageal squamous cell carcinoma cohorts**

Data for best overall response, objective response rate, and disease control rate, and duration of response are based on the efficacy evaluable set; data for progression-free survival and overall survival are based on the FAS. *95% CIs are based on Clopper-Pearson exact confidence interval. †Disease control rate=complete response+partial response+stable disease. ^§^Median and 95% CI are based on the Kaplan-Meier method. ^¶^Combined positive score is defined as the number of PD-L1-stained cells (tumor cells, lymphocytes, macrophages) divided by the total number of viable tumor cells, multiplied by 100. CI=confidence interval; EEAS=efficacy-evaluable analysis set; FAS=full analysis set; PD-L1=programmed death ligand 1; NE=not evaluable.

|  | GC/GEJ | | ESCC | |
| --- | --- | --- | --- | --- |
|  | PD-L1 CPS<5 | All | PD-L1 CPS <10 | All |
| EEAS, n | 11 | 19 | 7 | 20 |
| Objective response rate, n (%) | 3 (27.3) | 6 (31.6) | 1 (14.3) | 6 (30.0) |
| 95% CI* | (6.0-61.0) | (12.6-56.6) | (0.4-57.9) | (11.9-54.3) |
| Disease control rate, n (%)† | 9 (81.8) | 15 (78.9) | 4 (57.1) | 12 (60.0) |
| 95% CI* | (48.2-97.7) | (54.4-94.0) | (18.4-90.1) | (36.1-80.9) |
| Duration of response (months) | 4.4 | 4.3 | 8.7 | 8.7 |
| 95% CI* | 3.4, NE | (3.4-NE) | (2.8-NE) | (2.8-NE) |
| FAS, n | 11 | 20 | 7 | 20 |
| Progression-free survival^§^ |  |  |  |  |
| Median, months | 6.9 | 4.1 | 2.6 | 2.7 |
| 95% CI | (1.4-8.5) | (2.6-7.6) | (1.2-3.2) | (1.3-5.5) |
| Overall survival^§^ |  |  |  |  |
| Median, months | 15.0 | 13.7 | 12.0 | 10.4 |
| 95% CI* | (4.2-15.8) | (5.5-15.8) | (2.5-15.4) | (7.8-15.4) |

***Table 7*** Summary of TRAEs by preferred term (PT)

| Cohorts | GC/GEJ | | ESCC | | BTC | | Total | |
| --- | --- | --- | --- | --- | --- | --- | --- | --- |
| TRAEs^a^ by PT, n (%) | **All** | **Grade ≥3** | **All** | **Grade ≥3** | **All** | **Grade ≥3** | **All** | **Grade ≥3** |
| Proteinuria | 12 (60.0) | 0 | 9 (45.0) | 1 (5.0) | 9 (45.0) | 0 | 30 (50.0) | 1 (1.7) |
| Occult blood positive | 11 (55.0) | 0 | 6 (30.0) | 0 | 6 (30.0) | 0 | 23 (38.3) | 0 |
| Diarrhea | 9 (45.0) | 0 | 6 (30.0) | 0 | 6 (30.0) | 0 | 21 (35.0) | 0 |
| Blood bilirubin increased | 4 (20.0) | 0 | 7 (35.0) | 0 | 7 (35.0) | 1 (5.0) | 18 (30.0) | 1 (1.7) |
| Urinary occult blood positive | 6 (30.0) | 0 | 6 (30.0) | 0 | 6 (30.0) | 0 | 18 (30.0) | 0 |
| Blood thyroid-stimulating hormone increased | 4 (20.0) | 0 | 8 (40.0) | 0 | 5 (25.0) | 0 | 17 (28.3) | 0 |
| Asthenia | 8 (40.0) | 0 | 7 (35.0) | 0 | 2 (10.0) | 0 | 17 (28.3) | 0 |
| Anemia | 8 (40.0) | 2 (10.0) | 6 (30.0) | 0 | 2 (10.0) | 0 | 16 (26.7) | 2 (3.3) |
| White blood cell decreased | 8 (40.0) | 1 (5.0) | 6 (30.0) | 2 (10.0) | 1 (5.0) | 0 | 16 (26.7) | 3 (5.0) |
| Hypothyroidism | 4 (20.0) | 0 | 6 (30.0) | 0 | 5 (25.0) | 0 | 15 (25.0) | 0 |
| Hypertriglyceridemia | 4 (20.0) | 0 | 5 (25.0) | 1 (5.0) | 6 (30.0) | 1 (5.0) | 15 (25.0) | 2 (3.3) |
| Aspartate aminotransferase increased | 5 (25.0) | 1 (5.0) | 3 (15.0) | 1 (5.0) | 7 (35.0) | 0 | 15 (25.0) | 2 (3.3) |
| Appetite decreased | 4 (20.0) | 0 | 5 (25.0) | 0 | 6 (30.0) | 0 | 15 (25.0) | 0 |
| Hypertension | 4 (20.0) | 0 | 3 (15.0) | 2 (10.0) | 8 (40.0) | 2 (10.0) | 15 (25.0) | 4 (6.7) |
| Neutrophil count decreased | 6 (30.0) | 1 (5.0) | 6 (30.0) | 2 (10.0) | 1 (5.0) | 0 | 14 (23.3) | 3 (5.0) |
| Alanine aminotransferase increased | 5 (25.0) | 0 | 3 (15.0) | 0 | 4 (20.0) | 0 | 12 (20.0) | 0 |

*BTC* biliary tract carcinoma; *ESCC* esophageal squamous cell carcinoma; *GC/GEJ* gastric/gastroesophageal junction; *TRAE* treatment-related adverse event.

^a^Occurring in ≥20% of patients in all grades or in ≥5% of patients in grade ≥3 in the total population.

***Figure 1*: Trial profile**

Abbreviations: BTC, biliary tract carcinoma; ESCC, esophageal squamous cell carcinoma; GC, gastric; GEJ, gastroesophageal junction.


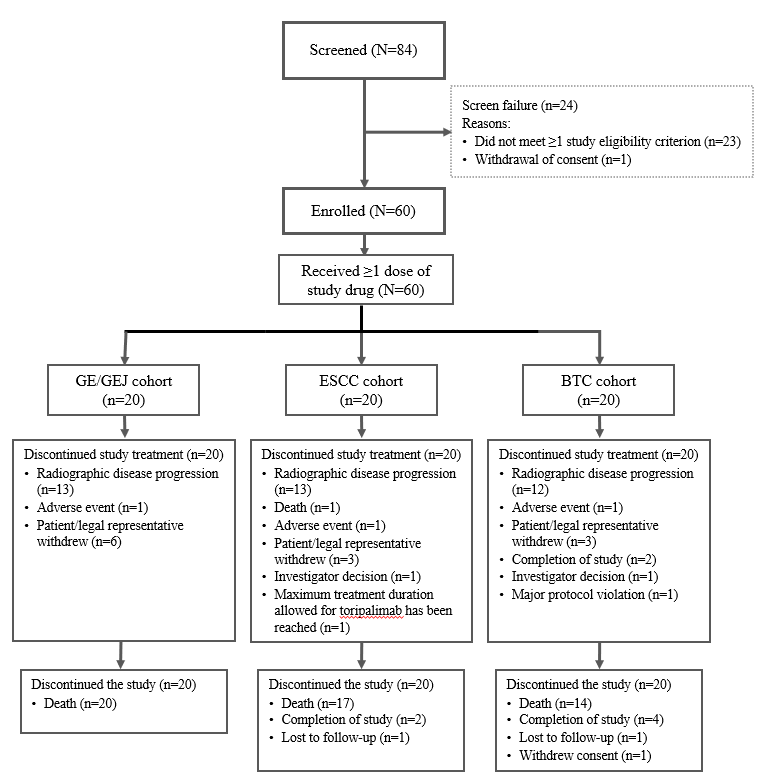

Supplement: Supplementary file 1 — Supplementary file1 (DOCX 168 KB) [file 262_2024_3677_MOESM1_ESM.docx]
